# Tracing Your Smart-Home Devices Conversations: A Real World IoT Traffic Data-Set

**DOI:** 10.3390/s20226600

**Published:** 2020-11-18

**Authors:** Marios Anagnostopoulos, Georgios Spathoulas, Brais Viaño, Javier Augusto-Gonzalez

**Affiliations:** 1 Department of Information Security and Communication Technology, Norwegian University of Science and Technology, 2815 Gjøvik, Norway; marios.anagnostopoulos@ntnu.no; 2 TELEVES S.A.U., 15706 Santiago de Compostela, Spain; bravia@televes.com; 3 Agata Technology, 15172 A Coruña, Spain; javier.augusto@agatatechnology.es

**Keywords:** IoT, smart-home, network, traffic analysis, data-set, security

## Abstract

Smart-home installations exponential growth has raised major security concerns. To this direction, the GHOST project, a European Union Horizon 2020 Research and Innovation funded project, aims to develop a reference architecture for securing smart-homes IoT ecosystem. It is required to have automated and user friendly security mechanisms embedded into smart-home environments, to protect the users’ digital well being. GHOST project aims to fulfill this requirement and one of its main functionalities is the traffic monitoring for all IoT related network protocols. In this paper, the traffic capturing and monitoring mechanism of the GHOST system, called NDFA, is presented, as the first mechanism that is able to monitor smart-home activity in a holistic way. With the help of the NDFA, we compile the *GHOST-IoT-data-set*, an IoT network traffic data-set, captured in a real world smart-home installation. This data-set contains traffic from multiple network interfaces with both normal real life activity and simulated abnormal functioning of the devices. The *GHOST-IoT-data-set* is offered to the research community as a proof of concept to demonstrate the ability of the NDFA module to process the raw network traffic from a real world smart-home installation with multiple network interfaces and IoT devices.

## 1. Introduction

Without a doubt, the Internet of Things (IoT) has attracted considerable attention during the last years, as the related technology presents a huge investment opportunity for many industrial and business stakeholders. It is estimated that by the end of this year a number of approximately 50 billion connected devices will be deployed and the total IoT revenue will outreach more than one trillion euros [1]. However, many agree that this forecast is already outdated.

During the recent years, the IoT systems growth has created significant security concerns as the number of the security incidents continuously multiply [2]. The most prominent example is the break out of the Mirai botnet, where the use of default authentication credentials allowed the cyber-crooks to take over hundreds of thousands of IoT devices with the purpose to entangled them in Distributed Denial of Service (DDoS) attacks [3]. As an emerging and low-cost technology, IoT is prone to cyber-security risks and thus the demand for safeguarding such ecosystem is constantly growing. Furthermore, the heterogeneity and diversity of the IoT devices, as well as the new lightweight communication protocols [4] suitable for the restricted resource and low-energy IoT installations, create new challenges for the protection of such systems.

Furthermore, smart-home appliances have become more appealing to the end-users and contribute to the emerging of a new market related to smart-home ecosystem. Physical security, healthcare/telecare applications, energy management and home entertainment are some of the most common concepts that smart-home installations offer to the end-users [5]. Given the aforementioned security concerns, the growing numbers of smart-home installations are highly vulnerable to cyber-security attacks and their protection is crucial for the end-users.

Towards this end, the GHOST—Safe-Guarding Home IoT Environments with Personalised Real-time Risk Control—project, a European Union Horizon 2020 Research and Innovation funded project, aims to develop a reference architecture for securing smart-homes IoT ecosystem [6]. It proposes a multi-layer solution that integrates traditional cyber-security countermeasures, while it introduces novel mechanisms for the efficient defence of common to IoT threats [7]. The main idea is that the network activity of the smart-home environment is monitored and fed to the various detection modules which analyze and decide about potential cyber incidents. In turn, the GHOST’s module responsible for the risk assessment evaluates the risk against the smart-home environment and either according to the user’s preferences or autonomously it proceeds to the mitigation measures for the identified risk.

As it is evident, the input of the GHOST solution, namely the raw network traffic, is of the utmost importance for the accurate and timely detection of the cyber-security events. In IoT networks and specifically in smart-home environments, a variety of low-energy, wireless, personal area network (PAN) protocols are deployed, each one utilizing different standards and carrying different fields and values. Due to the immaturity of the domain and the complexity of handling multiple different protocols, there is not any unified scheme to manage the traffic of the smart-home in a holistic approach.

The focus of the current paper is on NDFA, the module responsible for the analysis of the network traffic for the GHOST platform. Specifically, the NDFA module observes the network traffic for all the interfaces connected to the smart-home gateway, analyzes the captured traffic traces, and extracts valuable information. The remaining modules of the GHOST platform rely on the NDFA, in order to acquire meaningful info about the network traffic of the smart-home in an integrated format for all the heterogeneous protocols. In the context of the GHOST project, we also deploy the NDFA module in a real life smart-home installation to collect actual network traffic for the testing and evaluation of the GHOST solution. This network traffic is publicly offered as a proof of concept for the NDFA module functionality.

The contribution of the paper at hand is twofold:

- Firstly, we describe the GHOST’s module, called NDFA, responsible for the analysis of the raw network traffic and its initial processing that results in the extraction of meaningful data in the form of a compact data structure. The NDFA module can be utilized as a stand-alone application, whenever is required to analyze raw wireless network traffic of heterogeneous protocol within an IoT ecosystem.
- Secondly, we provide as a proof of concept the *GHOST-IoT-data-set* (https://github.com/gspathoulas/ghost-iot-dataset) to demonstrate the ability of the NDFA module to process the network traffic of a smart-home. This way, we offer to the research community a public data-set containing IoT network traffic collected from a real life smart-home installation. During the capturing, abnormal functioning of devices was intentionally triggered, to construct a data-set that enables the development of mechanisms appropriate for behavioral analysis of the activity within the smart-home.

The rest of the paper is organised as follows: Section 2 introduces the related work by presenting similar tools to NDFA, that contribute to the analysis and the extraction of data from IoT network traffic. Furthermore, this section reviews the most relevant IoT-based data-sets. Section 3 presents briefly the GHOST solution architecture, while Section 4 details on the implementation of our capturing and monitoring module. Following, Section 5 defines the data structure of the extracted information for each one of the considered wireless protocols. Section 6 describes the setup of the real life smart-home installation deployed for the collection and processing of the public data-set, documents the normal and abnormal activity in the home, and provides the analytics of the data-set. Finally, Section 7 concludes the paper with a discussion on the possible uses of the GHOST-IoT-data-set and draws possible future directions.

## 2. Related Work

### 2.1. Tools

With the proliferation of IoT technology, a plethora of tools were developed for the processing and feature extraction from the network traffic stemming from IoT devices. However, as we observe, the majority of them focus on the IP-based networks, and neglect to consider other wireless protocol. In a preliminary work, Xu et al. [8] developed a monitoring platform to capture network traffic from IP-based IoT devices within a smart-home network. They grouped the packets into flows and exported for each flow useful aggregated attributes such as source and destination IP addresses, ports, protocol, number of packets and bytes. Meidan et al. [9] dealt with the extraction of features from IoT network traffic in order to identify the type of the IoT device. They focused on TCP/IP traffic data as well, and they used solely packets from the TCP handshake session grouped according to the source and destination IP addresses and port numbers.

To put forward, Sivanathan et al. [10] extracted statistical attributes of the IoT devices based on their IP network traffic. Such attributes could help for the classification of the devices and their behavior. They studied the activity patterns of the devices as long as their signaling patterns. For the first case, they calculated the per flow attributes, such as flow volume, flow duration, average flow rate, and device sleep time. While for the later, they examined the utilization of ports, and the DNS and NTP queries. The authors acknowledged that these patterns could be used to determine the default baseline behavior of a given IoT device, which later on could facilitate the detection of abnormal behavior due to say a cyber-security event. A more related work to our monitoring tool is that of Calix et al. [11]. The authors developed a toolkit coded in Python for the pre-processing and feature extraction of data for cyber-security purposes, including the processing of network capture files from IoT devices. For the time being, the authors have examined the WiFi and TCP/IP protocol. The tool is able to extract the wireless link layer and IP network layer headers without the payload. In total, the tool extracts 21 features.

As it is evident from the previous discussion, the majority of the works that currently exist in the literature focus solely on the IP traffic, while they neglect to examine other wireless protocols. In our work, we follow a holistic approach, where we examine a variety of wireless network protocols, existing in an IoT environment. For these protocols, we extract the most essential fields of the packets and we calculate measurements of the traffic flows/connections. The considered wireless protocols are IP, including Ethernet, WiFi and PPP interface, Bluetooth, Z-Wave, RF869 and Zigbee.

### 2.2. Data-Sets

In the realm of data-sets containing wireless network traffic, the related research focus mainly on the 802.11 standard and for the purpose of training and evaluating intrusion detection methodologies. For intance, Kolias et al. [12] published a data-set, which they call the Aegean WiFi Intrusion Dataset (AWID), containing normal and attack traffic against a 802.11 network. Specifically, they implement a physical setup imitating a typical SOHO infrastructure, where they placed a number of mobile and stationary WiFi devices, like smartphones, laptops, desktop machines, tablet and a smart TV. In addition, they utilized a mobile attacker for launching the simulated attacks. The devices and the AP were communicating with WEP-protected 802.11 network. The simulated attacks were WEP specific and were categorized according to their methodology of execution of the injection, flooding, impersonation and passive attacks. The purpose of the data-set is to be used within the scope of intrusion detection in WiFi or in general in wireless networks.

Almomani et al. [13] produced a specialized data-set for Wireless Sensor Network (WSN) that contains normal and attack traffic simulating the DoS attack types of Blackhole, Grayhole, Flooding, and Scheduling attacks. They implemented the attacks against the Low Energy Aware Cluster Hierarchy (LEACH) routing protocol, one of the widely used routing protocols for WSNs environments. The WSN-DS data-set, as they call it, was collected with the usage of NS-2 simulator where the authors simulated a WSN network of 100 nodes and 5 clusters. The goal of the authors is to create a specialized data-set for training IDS for WSNs.

Similarly, Verma and Ranga [14] created a synthetic data-set for the case of the RPL routing protocol utilized in 6LoWPAN networks. This protocol is employed in IoT wireless networks. The so called RPL-NIDDS17 data-set was collected with the help of NetSim simulation platform. The authors simulated an IoT network with sensor nodes, gateway, router and wired nodes. The captured traffic was stored in CSV files, while they extracted 22 features representing flow, protocol connection and time attributes. The data-set, besides the normal traffic, contains also simulated traffic of routing attacks, such as sinkhole, blackhole, hello flooding, clone id, local repair, sybil and selective forwarding.

Al-Hadhrami and Hussain [15] made available the IoT-DDoS data-set. Specifically, they emulated in the Cooja simulation environment an IoT network consisting of 29 nodes, that imitated an industrial IoT environment. The nodes supported the IEEE802.15.4 standard and communicate with the 6LoWPAN protocol. The authors collected normal traffic as long as attack traffic simulating flooding, selective forwarding and blackhole attacks. From the captured network traffic, they extracted features from the physical layer, e.g., signal strength and transmission range, the network layer, e.g., data from RPL routing protocol and 6LoWPAN protocol, and the application layer, e.g., actual readings from the sensors. Apparently, the attack traffic affects the features related to the network layer and so these features are the most valuable of the data-set.

Sikeridis et al. [16] released the BLEBeacon data-set, which contains Bluetooth Low Energy (BLE) advertisement packets created by BLE beacons devices. The authors utilized stationary Raspberry Pi 3 (RPi) devices to collect the BLE advertisements within their university building, while the participants of the experiment were provided with a BLE beacon device to carry during their usual routines. The purpose of the data-set is to be used on location aware and human sensing applications. The data-set was collected over a period of one month.

More recently, researchers from Stratosphere Laboratory [17] released the IoT-23 labeled data-set that contains benign and malicious network traffic from IoT devices. For creating the benign traffic, the researchers utilized three real IoT devices, namely a Philips HUE smart LED lamp, an Amazon Echo home intelligent personal assistant and a Somfy smart doorlock. While for the case of malicious traffic, they use malware samples from well-known IoT botnet agents to infect Raspberry Pi devices. These devices utilized several network protocols and performed both malicious and legitimate actions. The utilized malware samples are bot agents for the Mirai, Hajime, IRCBot and other similar, which create network traffic for Command and Control (C&C) purposes as long as port scanning and attacking traffic. The purpose of the data-set is to facilitate the detection of IoT-based botnets.

Overall, for the case of smart-home related data-set, the research has focused mainly on the human activity [18], thus the actual measurements of the sensors or the detected events are collected rather than the network traffic. Efforts to standardize a universal format for storing and sharing data-set from smart-home environments do not take into account network characteristics [19].

From the previous discussion, we can see that the literature lacks an appropriate data-set of IoT traffic that will contribute to the IoT traffic analysis research. We aim to close this gap by offering to the research community the *GHOST-IoT-data-set*. Specifically, *GHOST-IoT-data-set* contains traffic from the normal and abnormal usage of the IoT devices, and it can be used for the training and the evaluation of behavioral analysis methods within a smart-home.

## 3. GHOST

IoT technology is prone to cyber-security risks due to the hardware heterogeneity, reduced processing capabilities making the use of complex algorithms infeasible, and the need of having solutions that are easy to deploy and use. Currently, there is a lack of a holistic mechanism that will protect IoT environments from cyber-security threats. In this context, GHOST has been focused on the development of a specific software solution for IoT environments, taking into account the restrictions of embedded devices, the need of adaptation to non-IP protocols, and the importance of non interfering with the deployment and normal operation of the services provided over the IoT network.

GHOST aims to develop a highly-affordable, turn-key, hassle-free solution for securing smart-home ecosystems from any form of cyber threat [6,7]. The solution relies on a central gateway that aggregates all the traffic from the smart-home network. Captured traffic traces may include variant local area IoT-oriented wireless protocols, such as WiFi (802.11), ZigBee (802.15.4) or Bluetooth, and wide area protocols, such as Ethernet or cellular network communications, that connect the smart-home environment with the Internet. In a nutshell, due to the GHOST solution, the smart-home’s gateway is able to capture all the ingress and egress traffic of the smart-home network. Furthermore, GHOST solution follows various approaches for cyber threat and risk detection by applying diverse cutting edge technologies, such as machine learning, intrusion detection and prevention, or blockchain. GHOST autonomously assesses the risk against the home network status and provides an intuitive and usable interface for the end-users to manage their security preferences and interventions.

In short, the architecture of the GHOST solution is composed by five layers:

- **Gateway layer**: This layer comprises of specific hardware and middleware implementations and offers the main mechanisms for accessing the hardware resources and for capturing the traffic that passes through the gateway. In addition, it provides the necessary functionality for operating over the interfaces, thus it enables actions like blocking or allowing specific connections or devices. In other words, this layer actually applies the security counter-measures for the supported protocols.
- **Data Interception and Inspection layer**: As explained later on, this layer is dedicated to extract and filter the network traffic captured by the gateway, which will be utilized for cyber-security analysis. Furthermore, it generates the required statistics for profiling the devices and the communication interfaces.
- **Contextual profiling layer**: This layer uses the output of the previous layer to perform statistical analysis for detecting deviations on the behavior of a device, a network interface or the user. Moreover, it integrates legacy cyber-security tools for IP networks, to detect IP-based attacks. Its purpose is to identify deviations that could constitute potential cyber-threats for the smart-home environment.
- **Risk assessment layer**: This layer integrates the outcomes of the various detectors to compute an overall risk for the smart-home environment. Based on the aggregated information from the underlying layers, it proceeds to autonomous decisions/actions or propagates notifications to the users and then acts according to their preferences. In addition, it integrates data from external knowledge sources, such as cloud repositories and blockchain infrastructure, that will contribute to the decision making process.
- **Control and monitoring layer**: This layer provides the end-user’s interface. It maintains the functionality for configuring the GHOST solution, for visualizing the status and the traffic of the network, and for managing the potential interventions for handling cyber-security-related incidents.

The GHOST system offers a holistic approach for the protection of smart-home environments from cyber-security threats. In detail, the Gateway layer provides the basic functionality that facilitates the interaction of the GHOST system with the hardware of the smart-home installation and thus the capturing of the network traffic. This traffic is analyzed by the Data Interception and Inspection layer, which converts the pcap files into an integrated representation for all the heterogeneous protocols. In turn, the Contextual profiling layer aims to build the behavioral profile of devices, interfaces and end-users based on several statistical metrics. The identified profiles could help to reveal deviations from the normal behavior due to cyber-incidents or more general misuse events. Following, the Risk assessment layer integrates the outcomes of the underlying detectors to calculate an overall risk of possible malicious events and according to the preferences of the users proceeds to autonomous mitigation actions or informs them about potential incidents. Finally, the Control and monitoring layer serves as the front-end interface to the user and provides the configuration and monitoring functionality in a user-friendly format, to enables smart home owners to more easily secures their installations.

**Figure 1:**
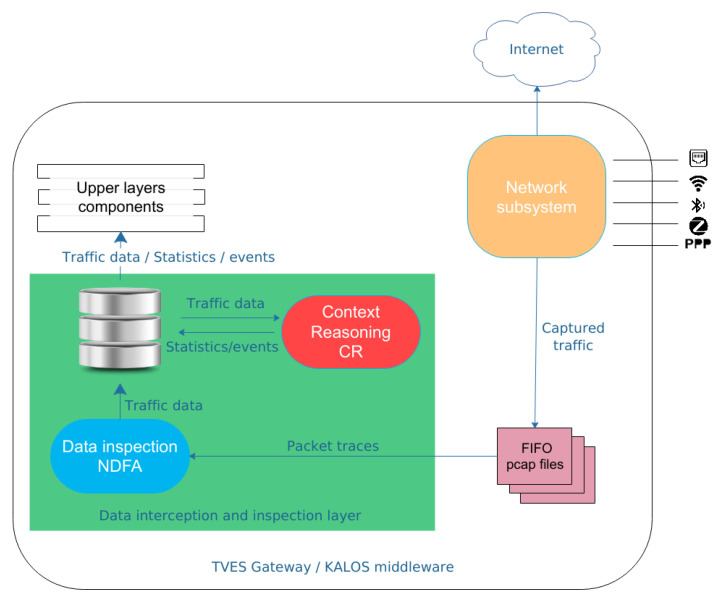
NDFA role in GHOST.

The GHOST platform is a hardware agnostic, software solution that can be installed on gateway devices, which usually have restricted resources. GHOST offers a combination of traditional cyber-security mechanisms with novel behavioral analytics approaches. The basis of this process is the Network and Data Flow Analysis (NDFA) component that monitors the traffic and extracts only the required data for each network protocol in an efficient and reliable way. Figure 1 illustrates the role of NDFA component in the GHOST platform. Specifically, the NDFA, as part of the Data Interception and Inspection layer, observes the network traffic for all the interfaces connected to the gateway, analyzes the captured traffic traces (pcap files), extracts the valuable information and finally stores them in the database [20]. Its functionality is critical for the rest of the GHOST components as it produces the main data flow on which those operate. The NDFA component’s functionality and how it has been used for the collection of the GHOST-IoT-data-set is the focus of the current paper.

## 4. Data Inspection Work-Flow

As mentioned already, the GHOST project’s main focus is to protect a smart-home installation from potential cyber-attacks. The main data source for the system is the network traffic passing through the smart-home gateway, which in turn is analyzed by the NDFA component.

The principal criteria for the design of NDFA are:

- Monitoring of all IoT related network protocols
- Compatibility with operating systems for embedded devices
- Minimal requirements in terms of libraries or installed software
- Efficient operation with minimal resources requirements
- Extraction of information on a per packet and on a per connection/session basis

The various communication protocols under consideration are those present in hardware installations used in the GHOST project’s pilots. These protocols are divided in IP protocols (PPP, Ethernet and WiFi) and non-IP protocols (Bluetooth, RF869, ZigBee and Z-Wave). The specifications and the packet structure for each of these protocols were studied, in order to determine the appropriate analysis approach for each case. In this Section, we describe the capturing and monitoring mechanism developed in the context of the GHOST system. Specifically, Section 4.1 discusses the capturing mechanism, Section 4.2 presents the approach used for the IP protocols and Section 4.3 describes the approach for the non-IP protocols.

For completeness, it is required to provide more details about the RF869 proprietary protocol utilized on this experiment. The main application of the deployment for this experiment is to provide telecare and telehealth services at smart-homes. In the standard that defines the provision of these services (EN-50134), it exists a dedicated radio frequency band that must be used for social alarm communication. This band has been defined as part of the Industrial, Scientific and Medical (ISM) bands by the ETSI and uses the frequencies between 869 and 869.25 MHz with a duty cycle of 0.1%. Due to the reduced bandwidth available, the modulation used in the RF869 protocol is a MSK modulation with data rates under 1 Kbps. The RF869 protocol consists of mainly two types of packets: system packets (intended for installation, monitoring and maintenance processes, like keep alive packets) and end-user related packets (intended to communicate alarms like the pressing of an emergency button or the activation of a gas detector). The length of the RF869 packets is between 7 (in keep alive packet) and 15 bytes (packets used to install devices).

### 4.1. Data Capturing

For all the monitored protocols, it was developed a capturing mechanism that reads raw traffic on the interfaces and generates sequential non-overlapping pcap files, as the network traffic is captured consecutively. For this purpose, several circular buffers are implemented within the gateway, one per each communication protocol. Each one of the buffers is composed by several pcap files. The buffers are determined by the maximum number of traffic traces that they can handle, the maximum total size in bytes of the buffer, the maximum size in bytes of each of the traffic traces and the maximum time duration of each trace. The flexibility for independent configuration of the maximum size and maximum duration for each protocol enables to adjust the capturing mechanism to operate near real-time with different protocols. For instance, for protocols that exhibit low data rate and scattered communication, the maximum duration parameter will keep the generation of new network traces stable and continuous, while in the case of protocols with high data rates and continuous communication, the maximum size parameter will prevent from the overwhelming of the gateway’s storage capabilities.

**Figure 2:**
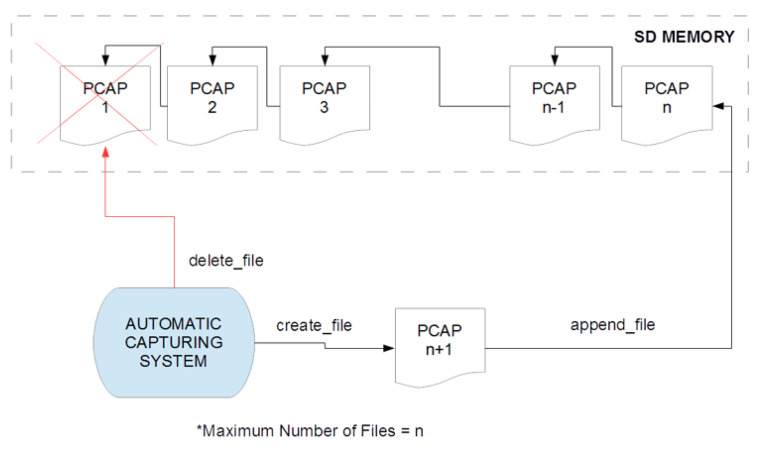
Circular capturing buffers implemented for each communication protocol.

As depicted in Figure 2, when the maximum number of pcap files is reached, the oldest one is automatically discarded and a new one is appended to the buffer.

As already mentioned, the capturing process is implemented in the gateway. Specifically, the tshark library is utilized for the capture of the IP protocols traffic and Bluetooth traffic. For the case of Zigbee (802.15.4) interface, a specialized network integrated circuit of Texas Instrument with a Zigbee Network Processor (CC2531) is used. With the help of this circuit, Zigbee packets are reconstructed in the application layer to create the pcap file with the packets in the 802.15.4-format. Furthermore, the gateway also offers a proprietary interface on 869MHz ISM band for telecare communication purposes, called RF869. An ad-hoc capturing and pcap generation software has been developed for RF869 proprietary interface and Z-Wave interface following the aforementioned approach of Zigbee.

### 4.2. IP Traffic Work-Flow

**Figure 3:**
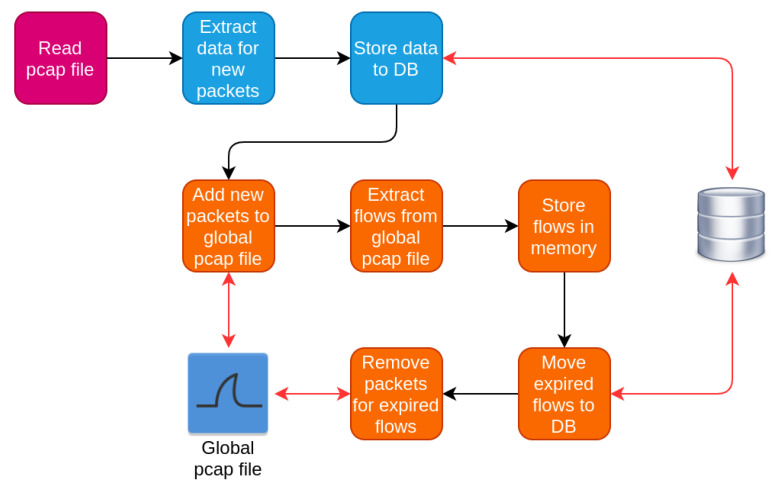
Work-flow of data inspection subsystem for IP traffic.

The work-flow of the data monitoring subsystem is an iterative process for the IP traffic, as illustrated in Figure 3. The black edges indicate the actual workflow, while the red ones indicate data exchange between the system and the data storage resources such as the database or the pcap files. The captured traffic is analyzed initially on a per packet and then on a per flow basis, through which packets are grouped per communication session. The outcome of this analysis is information for both packets and flows. The exact structure for each packet may differ depending on the transport or application protocol. The main steps for the analysis of the IP traffic are:

- Read the current pcap file with the network traffic of IP interface.
- Extract the packet details from the new packets and store them to the database.
- Insert new packets to global pcap file maintained by the component. This file contains traffic for the active flows.
- Process the global pcap file to extract flows and store data for extracted flows temporarily in memory.
- Examine whether the extracted flows are still active or have expired.
- Store the data for expired flows permanently to database.
- Discard packets of expired flows from the global pcap files.
- Start over

#### 4.2.1. IP Packets Analysis

For the case of the IP traffic analysis, the packets are processed with the help of the dpkt Python library [21]. This library reads each individual packet from the pcap file and extracts its contents. The extracted information includes important fields from the packet header along with metrics, such as the packet’s size. The exact scheme of the retrieved information depends on the transport or application layer protocol of the packet, so even for the same network interface it is not feasible to define a default data structure. The extracted data fields are summarized in Section 5.2.1.

#### 4.2.2. IP Flows Analysis

**Figure 4:**
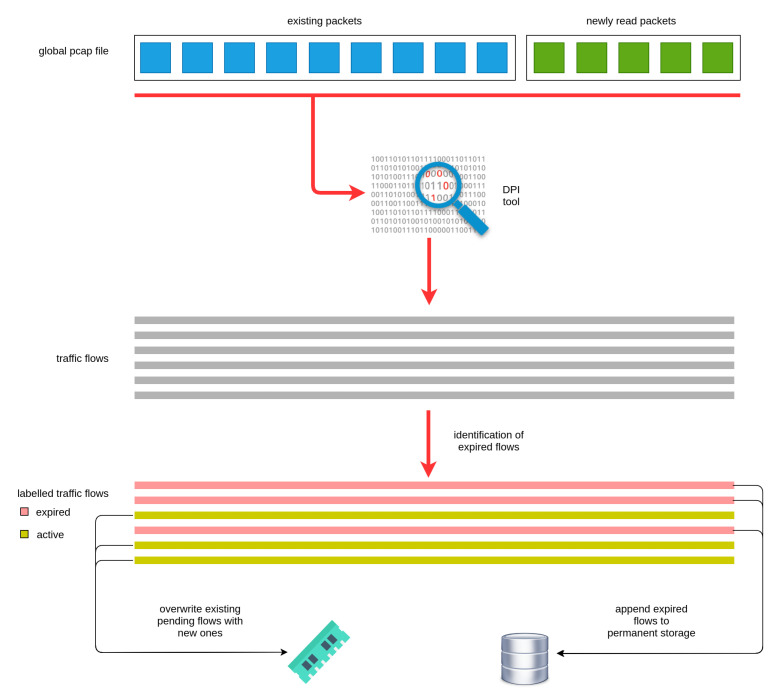
Traffic flows management.

Furthermore, the monitoring module extracts the traffic flows consisting of groups of packets related to the same transaction. However, these packets may expand in more than one pcap files. Thus, the component maintains a global pcap file, where it stores the packets for all active traffic flows. There, it appends new packets after each new pcap file is read. The top part of Figure 4 illustrates how the global pcap file is updated with the new (green) packets.

Afterwards, the global pcap file is fed to the *Libprotoident* DPI tool [22], which outputs the traffic flows along with relevant data and statistics. However, due to the iterative nature of the pcap file process, it is essential to monitor the flows at each iteration and classify those as either active or expired. In the case of active flows, these are stored temporarily in memory, while in the case of expired flows, they are stored permanently to the database. As shown in Figure 4, the module has detected expired flows depicted in red colour, while the active ones are depicted in yellow colour.

For deciding whether a flow has expired, the module examines the time duration that the traffic flow is idle, namely it checks the elapsed time since the last captured packet of the flow. In the case, that this time is larger than the predefined threshold value, then the flow is marked as expired. Otherwise, the flow is marked as active. The bottom part of Figure 4 denotes the process of storing the flows in the proper storage resource. Furthermore, in each iteration the global pcap file is trimmed based on the expired flows and the corresponding packets are discarded.

### 4.3. Non-IP Protocols Work-Flow

**Figure 5:**
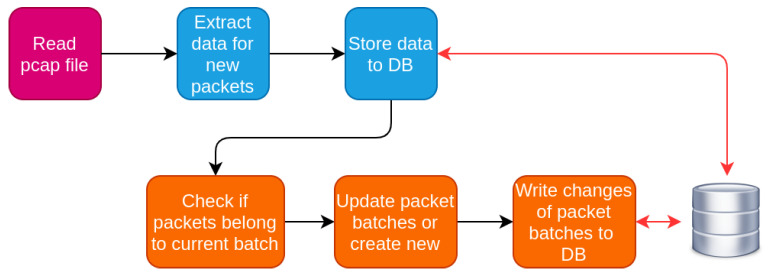
Work-flow for non-IP protocols.

The traffic processing workflow for the non-IP protocols is similar to that of the IP traffic, as depicted in Figure 5. Although, the packet analysis procedure follows the same steps as in Section 4.2.1, the grouping of packets is differently implemented, because of the distinction between IP and non-IP protocols. Specifically, in the non-IP protocols the packets are grouped in batches, which are dynamically built upon packets’ distribution in time.

#### 4.3.1. Packet Analysis

The process of reading the sequential pcap files, extracting data for new packets and storing this data to the database is akin to the corresponding process for the IP traffic case, as described in Section 4.2.1. Technically, the pcap files for the non-IP protocols are processed by the *tshark* [23] tool and specifically its Python wrapper *pyshark* [24].

#### 4.3.2. Batches Analysis

The flows approach used in the case of IP traffic is inappropriate to the case of the non-IP interfaces. In the majority of these protocols, there is no established session between two nodes. In most cases each packet is handled as a single independent communication event. Additionally, for non-IP protocols the traffic is characterized by:

- Reduced number of packets.
- Sparse number of concurrent connections.
- Less packets with actual payload.
- More packets carrying events or commands (management packets).

By examining the traffic traces for the non-IP protocols, we observed that the exchanged packets between distinct pairs of communicating devices form clusters in time. Thus, we assumed that such clusters belong to the same high level action or event. We concluded that the most efficient approach to detect such clusters was to set a fixed time window in order to determine when a batch of packets is completed. In reality, the majority of the produced batches were short identical series of packets related to specific recurring actions. Therefore, we group the packets for the non-IP protocols to batches based on their timestamp and the pair of communicating devices.

**Figure 6:**
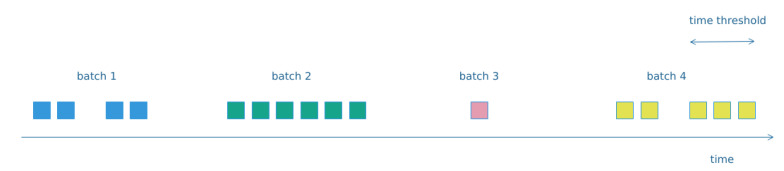
Building packet batches for Non-IP protocols.

Moreover, for the case of the Bluetooth interface, besides the packets containing the actual data from the measurements (payload), we observed also the exchange of many control packets. Such messages are related with broadcasting or keep alive messages and are usually unrelated with the activity occurring in the smart-home. Their frequency is significantly higher than that of the data packets, and this fact hindered the formation of batches related to actual events/actions. However, we expect to detect evidence of cyber-security attacks in the control packets as well, so discarding them, it is not appropriate. For this reason, we concluded to label the Bluetooth packets as either data or management and handle those two categories separately, when constructing the batches. The followed approach is depicted in Figure 6.

## 5. Smart-Home Traffic Data Scheme

In this section, we detail on the scheme of every network interface under consideration. As mentioned in Section 4, the format of the output is related to both the monitored interface and the protocol of the communication.

### 5.1. Tables

The output of the monitoring component does not follow a predefined structure due to the diversity of the packet type and fields. Overall, for each interface there are two tables, these are on packet level and on flow/batch level. Both tables store an id and a JSON field containing the data for the specific packet or flow/batch. The JSON field contains a set of a key-value pairs, one for each parameter extracted. The JSON’s field format is not static and depends on the protocol of the communication. However, this format facilitates the retrieval of the fields by querying the values of every fields or of a subset of them. The considered protocols are IP (including Ethernet, WiFi, and PPP), Bluetooth, Z-Wave, RF869 and Zigbee, thus the utilized tables are:

- **ip_packets:** One record per captured IP packet.
- **ip_flows:** One record per captured IP flow.
- **bt_packets:** One record per packet captured on the Bluetooth interface.
- **bt_batches:** One record per batch captured on the Bluetooth interface.
- **zw_packets:** One record per packet captured on the Z-Wave interface.
- **zw_batches:** One record per batch captured on the Z-Wave interface.
- **rf869_packets:** One record per packet captured on the RF869 interface.
- **rf869_batches:** One record per batch captured on the RF869 interface
- **zgb_packets:** One record per packet captured on the Zigbee interface
- **zgb_batches:** One record per batch captured on the Zigbee interface

### 5.2. IP Traffic Data Format

As concerns the IP traffic, it may be related to the Local Area Network (LAN), namely Ethernet, the Wireless Local Area Network (WLAN), namely WiFi, or PPP interface of the smart-home gateway. Furthermore, the IP traffic might carry packets from different transport layer.

#### 5.2.1. Packet Analysis

Independent of the packet’s transport layer protocol, there are some common fields extracted from the its IP header, which are listed below:

- **Source IP:** The source IP of the packet in either IPv4 or IPv6 format.
- **Destination IP:** The destination IP of the packet in either IPv4 or IPv6 format.
- **Source MAC:** The physical MAC address of the source host. Not applicable to PPP protocol.
- **Destination MAC:** The physical MAC address of the destination host. Not applicable to PPP protocol.
- **Identification:** The unique id of the IP packet.
- **Length:** The length of the IP packet.
- **Timestamp:** The packet’s timestamp in epoch format.
- **Transport Protocol:** The transport protocol of the packet, e.g., TCP, UDP, ARP, ICMP.
- **Data:** The payload of the IP packet.

Regarding the transport layer, the TCP and UDP transport protocol are examined. Regarding TCP protocol, the source and destination ports, the length of both the header and the payload, and the most important of the flags values are extracted. These fields are:

- **Source port:** The source port of the packet.
- **Destination port:** The destination port of the packet.
- **Flags:** The flags of the TCP header.
- **Options:** The options of the TCP header.

While, in the case of the UDP protocol, solely the source and destination ports and the length of the payload are stored, as shown below:

- **Source port:** The source port of the packet.
- **Destination port:** The destination port of the packet.

For the remaining protocols, like ICMP, ARP etc., only the fields denoted for IP packets are stored to the database.

#### 5.2.2. Data Flows Analysis

As already explained in Section 4.2.2, the IP traffic is structured into flows. Each flow corresponds to a session between the two hosts. Such flows are constructed from the pcap files and NDFA outputs the related data, along with valuable statistical metrics. The data for each flow are sorted into static and dynamic data. The static data are calculated from the fields and payload of the packets contained in the flow. These data are:

- **IP address of A:** The IP address of the first endpoint of the flow, that is the sender of the first packet of the flow.
- **IP address of B:** The IP address of the second endpoint of the flow, that is the receiver of the first packet of the flow.
- **MAC of A:** The MAC address of the first endpoint of the flow, that is the sender of the first packet of the flow. Not applicable to PPP flows.
- **MAC of B:** The MAC address of the second endpoint of the flow, that is the receiver of the first packet of the flow. Not applicable to PPP flows.
- **Port of A:** The port used by the first endpoint of the flow.
- **Port of B:** The port used by the second endpoint of the flow.
- **Starting timestamp:** The timestamp of the first packet of the flow.
- **Stopping timestamp:** The timestamp of the last packet of the flow.
- **Application protocol:** The application protocol of the communication. Possible values are DNS, FTP, HTTP, IMAP, etc.
- **Transport protocol:** The transport protocol of the connection, possible values are either 6 for TCP or 17 for UDP.
- **First 4 bytes from A:** The first payload’s four bytes of the first packet send by A.
- **First 4 bytes from B:** The first payload’s four bytes of the first packet send by B.
- **Size of first packet sent from A:** The size of the first payload-bearing packet sent by the first endpoint.
- **Size of first packet sent from B:** The size of the first payload-bearing packet sent by the second endpoint.

On the contrary, the dynamic data are calculated from the entire duration of the flow and include various traffic metrics. This also incorporates statistics regarding the size of packets or their inter-arrival times. These metrics are useful for behavioral analysis, as they may reveal deviations from normal network traffic patterns. The dynamic data fields are shown below:

- **Packets from A:** Total number of packets sent from the first endpoint to the second endpoint.
- **Bytes from A:** Total number of bytes sent from the first endpoint to the second endpoint.
- **Packets from B:** Total number of packets sent from the second endpoint to the first endpoint.
- **Bytes from B:** Total number of bytes sent from the second endpoint to the first endpoint.
- **Min payload from A:** Minimum payload size sent from first endpoint to second endpoint.
- **Mean payload from A:** Mean payload size sent from first endpoint to second endpoint.
- **Max payload from A:** Maximum payload size sent from first endpoint to second endpoint.
- **Standard deviation payload from A:** Standard deviation of the payload size sent from first endpoint to second endpoint.
- **Min payload from B:** Minimum payload size sent from second endpoint to first endpoint.
- **Mean payload from B:** Mean payload size sent from second endpoint to first endpoint.
- **Max payload from B:** Maximum payload size sent from second endpoint to first endpoint.
- **Standard deviation payload from B:** Standard deviation of the payload size sent from second endpoint to first endpoint.
- **Min inter-arrival from A:** Minimum packet inter-arrival time for packets sent from first endpoint to second endpoint.
- **Mean inter-arrival from A:** Mean packet inter-arrival time for packets sent from first endpoint to second endpoint.
- **Max inter-arrival from A:** Maximum packet inter-arrival time for packets sent from first endpoint to second endpoint.
- **Standard deviation inter-arrival from A:** Standard deviation of the packet inter-arrival time for packets sent from first endpoint to second endpoint.
- **Min inter-arrival from B:** Minimum packet inter-arrival time for packets sent from second endpoint to first endpoint.
- **Mean inter-arrival from B:** Mean packet inter-arrival time for packets sent from second endpoint to first endpoint.
- **Max inter-arrival from B:** Maximum packet inter-arrival time for packets sent from second endpoint to first endpoint.
- **Standard deviation inter-arrival from B:** Standard deviation of packet inter-arrival time for packets sent from second endpoint to first endpoint.

### 5.3. Non-IP Traffic Data Format

The traffic analysis for the Non-IP protocols is similarly conducted on a per packet and a per batch level.

#### 5.3.1. Packet Analysis

##### Bluetooth

Bluetooth specification defines four packet types:

- HCI Command packets
- HCI ACL data packets
- HCI Synchronous data packets
- HCI Event packets

For all of these types, there are several common fields extracted, while additional fields are extracted according to the type of the packet. The common fields are:

- **Type:** An integer value corresponding to the type of the packet, specifically 1 corresponds to HCI Command, 2 to HCI ACL data, and 4 to HCI event packets.
- **Direction:** Defines the direction of the packet. Packets sent by the gateway are denoted as 0, while packets received by the gateway as 1.
- **Time:** The packet’s timestamp in epoch format.
- **Length:** The packet’s length in bytes.
- **Taxonomy:** Indicates whether the packet is management or data related, it takes two values, i.e., man and data.

The main fields extracted for HCI Command type packets are the codes related to the carried command and the length of the parameters embedded in the packet. There are three codes related to the command, which define the group it relates to and its functionality. These fields are:

- **Opcode:** An integer corresponding to the command of the packet. It is built by a combination of the two following codes and it is unique for each command throughout the protocol.
- **Opcode_ogf:** An integer corresponding to the group of the command. It is unique for each command subgroup and can be used to identify the general category of the command.
- **Opcode_ocf:** An integer corresponding to the command of the packet. It is unique for each command only throughout its subgroup. If a command should explicitly identified, then this code is not appropriate as duplicate opcode_ocf may arise between commands of different subgroups.
- **Parameters length:** The length of all the parameters measured in bytes.

When it comes to ACL data packets, the additional fields extracted are related to the source and destination addresses, along with the length of the packet’s data. These are:

- **Source address:** The source address of the packet.
- **Destination address:** The destination address of the packet.
- **Data length:** The length of the packet’s data in bytes.
- **Opcode:** The method of the Bluetooth Attribute Protocol.
- **Service:** The Service UUID of the method of Bluetooth Attribute Protocol.
- **Value:** The value of Bluetooth Attribute Protocol.

In the case of HCI event packets, there exist a variability regarding the fields present in each packet, which depends on the corresponding type of event. The monitoring scheme considers only the event code of the packet:

- **Event code:** The code related to the different types of events.

##### Z-Wave

For each Z-Wave packet, the extracted fields are the timestamp, source and destination addresses, length of data and the actual payload of the packet, as detailed below:

- **Source address:** The source address of the packet.
- **Destination address:** The destination address of the packet.
- **Timestamp:** The timestamp of the packet.
- **Data length:** The length of the packet’s data in bytes.
- **Data:** The actual payload data of the packet.
- **Text:** The collection of the key-value parameters in the payload of the packet represented as text.

##### RF869

The extracted fields for the case of RF869 packets are the timestamp, the address of the device that communicates with the gateway, the type of the packet, the length of the payload, as well as the actual payload of the packet. Note that the RF869 packets contain only the address of the communicating device, while that of the gateway is omitted.

- **Device address:** The address of the device communicating with the gateway.
- **Timestamp:** The timestamp of the packet.
- **Type:** A bitmap that describes the functionality of the packet.
- **Data length:** The length of the packet’s payload in bytes.
- **Data:** The application data of the packet (may be empty).

##### ZigBee

The fields for the ZigBee packet are the timestamp, source and destination address, the destination personal area network (PAN) id, the length of the packet and of the payload in bytes, and the raw data of the payload.

- **Source address:** The source address.
- **Destination address:** The destination address.
- **Destination PAN id:** The destination PAN id.
- **Timestamp:** The timestamp of the packet.
- **Packet length:** The length of the packet in bytes.
- **Data length:** The length of the packet’s payload in bytes.
- **Data:** The raw data of the payload.

#### 5.3.2. Packet Batches Analysis

As explained in Section 4.3.2, packets were aggregated into batches by grouping on the timestamp and the pair of communicating devices. Each produced batch corresponds to a specific action/event within the smart-home.

##### Bluetooth

In the case of Bluetooth protocol, two types of batches are created. The first type contains solely management packets (HCI Command or HCI event packets). Those packets do not carry addresses and are grouped only according to their timestamp. The second type contains data-related packets and are grouped according to the timestamp and the pair of the communicating entities. For each one of the detected batches, we calculate a series of metrics for the behavioral analysis of the traffic.

The fields for management batches are:

- **Start time:** The timestamp of the first packet of the batch stored in Linux time epoch format.
- **Stop time:** The timestamp of the last packet of the batch stored in Linux time epoch format.
- **Duration:** The time duration of the batch in seconds.
- **Taxonomy:** Indicates whether the batch contains management or data related packets, it takes two values, i.e., man and data.
- **Number of packets:** The total number of packets contained in the batch.
- **Minimum size:** The length in bytes of the smallest packet in the batch.
- **Maximum size:** The length in bytes of the largest packet in the batch.
- **Average size:** The average length in bytes of all the packets in the batch.
- **Total sum:** The total sum of the bytes of packets contained in the batch.
- **Batch id:** A string representing the series of the types of packets in the batch.

Special mention is required for the last field, Batch id, which is a string that is built from the integers representing the type of the packet in the series of packets within the batch. It is observed that this id is characteristic for each event, as the series of packets exchanged is strictly defined.

The following list describes the additional fields for the batches that consist of the data related packets.

- **Source address:** The address of the first endpoint of the batch.
- **Destination address:** The address of the second endpoint of the batch.
- **Packets from A:** Total number of packets sent from the first endpoint to the second endpoint.
- **Bytes from A:** Total number of bytes sent from the first endpoint to the second endpoint.
- **Packets from B:** Total number of packets sent from the second endpoint to the first endpoint.
- **Bytes from B:** Total number of bytes sent from the second endpoint to the first endpoint.

##### Z-Wave, Zigbee and RF869

The packets captured on the Z-Wave, Zigbee and RF869 interfaces are grouped in batches according to their timestamp and the addresses of communicating devices. The batches’ fields are described in the following list. Those are identical between the three interfaces with the difference that RF869 batches contain one instead of two addresses.

- **Source address:** The source address of the first packet. This address is used as part of the key for grouping the batch.
- **Destination address:** The destination address of the first packet. This address is used as part of the key for grouping the batch. Not applicable for RF869 interface.
- **Start time:** The timestamp of the first packet of the batch stored in Linux time epoch format.
- **Stop time:** The timestamp of the last packet of the batch stored in Linux time epoch format.
- **Duration:** The time duration of the batch in seconds.
- **Number of packets:** The total number of packets contained in the batch.
- **Minimum size:** The length in bytes of the smallest packet in the batch.
- **Maximum size:** The length in bytes of the largest packet in the batch.
- **Average size:** The average length in bytes of all the packets in the batch.

As concerns the selected fields extracted for each of the protocols, there were two considerations. The followed approach is to record the header’s fields and the payloads of the packets which are important in relation to the detection of anomalous behavior in a smart-home system. Due to the fact that the monitoring mechanism will be deployed on devices with restricted computational resources, it is also crucial that the extraction of the information should be accomplished with the minimum possible requirements. Therefore, the final selection of the fields was based on a combination of information that can facilitate the detection of multiple cyber-security incidents.

For instance, a DoS attack is anticipated to significantly increase the volume of the exchanged packets and decrease the packets’ inter-arrival time in the case of IP protocol. Similarly, a device impersonation attack will considerably alter several of the characteristics of the non-IP protocols batches like the duration, the number of packets and batch id of the batches originating from the impersonated device. Moreover, sleep deprivation attacks aiming to exhaust the battery of IoT devices will certainly create new packet batches which will be indicative of the ongoing activity. Finally, any malicious code injection in an IoT device will alter the data reported from the device, thus this incident can be detected by a payload anomaly detection mechanism applied on non-IP protocols.

## 6. Real World Smart-Home Data-Set

The aforementioned monitoring scheme has been deployed in the scope of a real-world experiment, as presented in this Section. The aim of the experiment is to collect a real-world data-set containing smart-home traffic, with the purpose of classification training. The traffic is collected for a period of ten days in a medium sized smart-home and contains traffic generated from both normal and abnormal use of the IoT devices. Specifically, during the first nine days of the capturing period, the smart-home inhabitants behave normally and the corresponding traffic traces reflect the normal status of the system. During the last day, seven abnormal scenarios are triggered either through intentional actions or due to simulation of a device’s malfunction. The produced data-set is used in the context of the GHOST project to enable the training of the system’s components, regarding the abnormal activity detection in the smart-home environment.

The generated pcap files are fed to the NDFA component in order to extract the useful data out of the network traffic. The outcome of the NDFA component containing all the packet and flows/batches records during the 10-day experiment is publicly available to the research community in the form of csv files. Bear in mind that in order to protect the privacy of the volunteer participants, we anonymized the fields that may contain sensitive and identifiable information. Specifically, we apply a HMAC function to the values of the address fields for each communicating devices, namely source and destination IP address, source and destination MAC address, source and destination address for the Bluetooth and ZigBee protocol, for both packets and flows/batches records. Furthermore, we excluded the payload for the IP-based packets.

It is worth to be noted, that the wireless network traces can reveal sensitive and identifiable information regarding the inhabitants of the smart-home to any eavesdropper. Indeed, any malicious actor in the vicinity of the home premises is capable of capturing the wireless network traffic and compromise the user’s privacy [25]. From our side, the capturing and analysis of the wireless network traffic takes place in the gateway, while no other external and irrelevant to the smart-home device is deployed. This way, we aim to limit the exposure of the sensitive and identifiable data by using them only within the smart-home installation and only for the purpose of improving the inhabitants’ security. Furthermore, we do not utilize any external or third-party service and application, that could possibly transfer the private data outside the smart-home ecosystem.

### 6.1. Environment

The smart-home environment is a single-family residence (approximately 105 square meters) with two (a man and a woman) inhabitants and a pet (a cat). Both inhabitants are between 30 and 35 years old and are actively working, meaning that there are many periods of time without presence in the house. Specifically, during the weekdays there is no presence in the home the period between 08:30 to 14:00 and 15:20 to 20:30.

**Figure 7:**
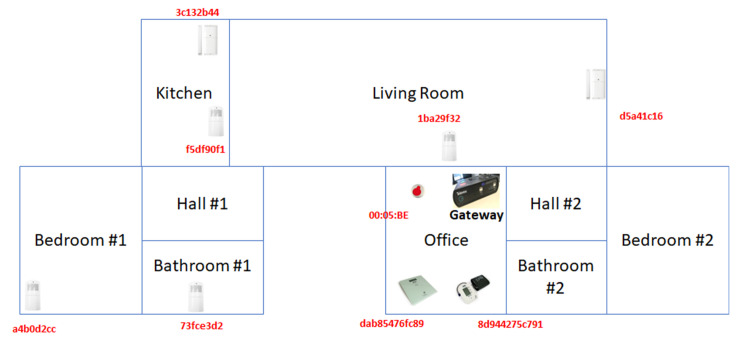
Plan of the smart-home installation.

Figure 7 illustrates the smart-home installation by depicting the layout of the installed IoT devices on the plan of the residence. The distribution of the IoT devices imitates the typical installation of a tele-care environment, while motion and door sensors are used for home security.

### 6.2. Installation Description

The gateway of the smart-home installation offers the following network interfaces:

- **802.11 (Wifi)**: The main purpose of the WiFi interface is to allow the user to connect to the gateway from a handheld device in order to monitor the connectivity status of the gateway with the IoT devices and to observe the application level data.
- **Ethernet**: The Ethernet interface is mainly utilized to monitor the internal processes of the gateway and to extract the pcap files from the internal memory of the gateway through SSH connection. The captures of this interface are not directly related to the activity in the smart-home.
- **PPP**: This interface is used by the gateway to access the Internet, for instance to access NTP service, connect with update servers, etc.
- **Bluetooth**: The Bluetooth interface allows the connection and exchange of data with the Bluetooth devices installed in the smart-home.
- **802.15.4 (ZigBee)**: The ZigBee interface allows the connection and exchange of data with the 802.15.4 capable interface.
- **RF869**: RF869 is a proprietary wireless interface that allows the connection and exchange of data with the RF869 devices installed in the smart-home.

Multiple IoT devices, of various protocols, are installed in the monitored smart-home. Table 1 presents these IoT devices, the smartphones and the personal computers that interact with the gateway during the capturing period. Bear in mind, that the smartphone is mainly used for monitoring purposes, while the laptop is connected to the gateway via Ethernet connection to monitor the internal processes of the gateway and copy the captured pcap files via an SSH connection.

### 6.3. Capturing

The capturing process begins after finalising the configuration of the environment. The system is set to a capturing-only configuration, generating a set of pcap files without the use of circular buffer control as explained in Section 4.1. The capturing process takes place during the period of 10 October 2019 (Thursday) afternoon and 20 October 2019 (Sunday) afternoon.

During this period, the inhabitants use the Bluetooth measurement devices at least once per day while the ZigBee sensors (motion sensors and door opening sensors) are triggered multiple times per day as they are installed in places visited regularly by the inhabitants. The RF869 button is not activated during the normal capturing period. During the last day of the experiment, seven different abnormal behavioral scenarios are executed, in order to simulate device malfunctioning or malicious activity by an attacker. These scenarios include patterns of consecutive measurements (emulating an attack aiming to flood the system with many successive measurements), removal of the batteries (emulating physical battery drain or firmware modification attacks), connection of unknown devices and relocation of the sensors throughout the home. All the aforementioned actions are presented in detail in Table 2.

The resulting pcap files are fed to the monitoring mechanism described in Section 4. The produced packets and flows/batches records compose the GHOST-IoT-data-set. This data-set reflects the normal usage of the environment for the first nine days, while the last day includes the different types of abnormal activity in the smart-home. The data-set is used in the context of the GHOST project, to validate the efficiency of the GHOST system’s components in detecting abnormal activity within the smart-home environment.

### 6.4. Data-Set Analytics

The collected data-set is the outcome of the activity in the smart-home. It contains data from the six different interfaces present in the gateway during the data collection and is organized into two distinct levels of abstraction, namely packets and flows/batches. Specifically, the six interfaces are Ethernet, WiFi, PPP, Bluetooth, Zigbee and RF869, and the statistics of packets/flows/batches are depicted in Table 3.

As it is observed, PPP is the most traffic loaded interface, since it is used for the communication of the gateway with the Internet. WiFi interface is used for accessing the gateway’s web interface, so it contains less traffic and finally Ethernet is only used to check the internal processes of the gateway, so it contains very limited traffic. Regarding the non-IP protocols, Bluetooth traffic consists of a large volume of packets, but most of them are related to the management traffic. Zigbee protocol presents some traffic which is characterized by short batches (2.7 packets per batch) and RF869 traffic contains mainly keep alive packets as no RF869 devices were activated except on the last day (1.01 packets per batch).

To depict the activity for each interface, we include a set of figures that represent the time distribution of the packets for each interface. Each figure illustrates the number of packets per time slot for the whole capturing period. A 4-hours slot is used to depict the activity in the smart-home in an aggregated form.

**Figure 8:**
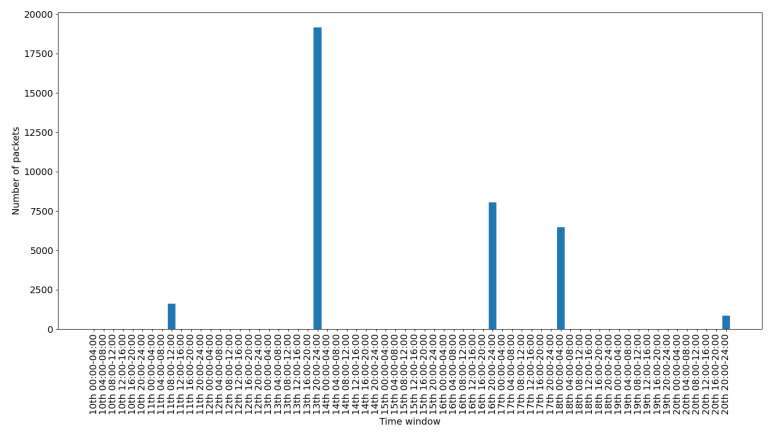
Ethernet packets time distribution.

**Figure 9:**
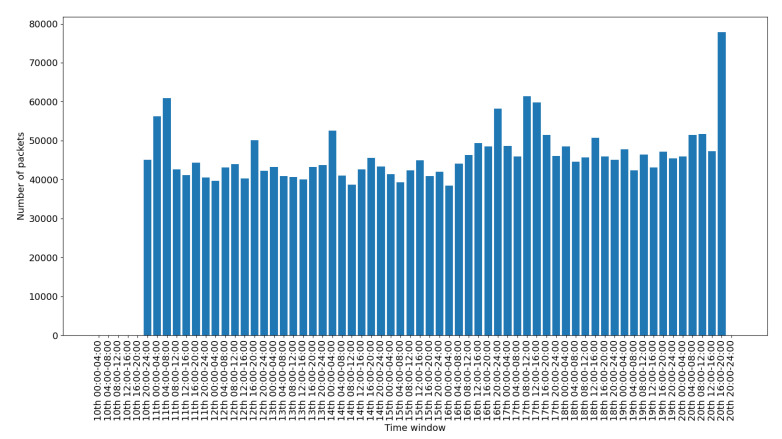
PPP packets time distribution.

**Figure 10:**
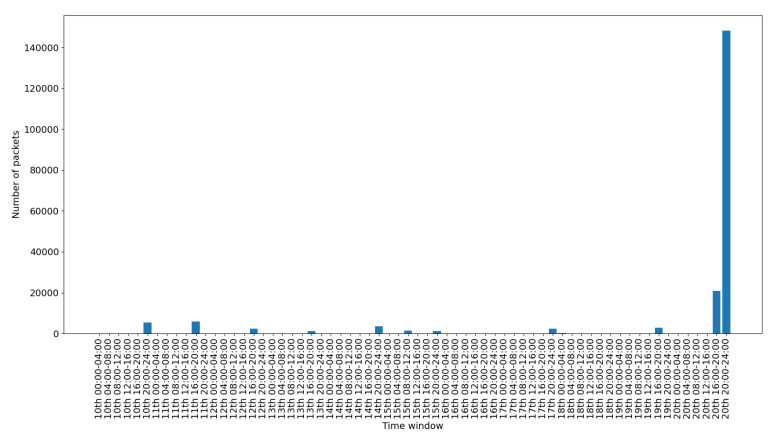
Wifi packets time distribution.

Figure 8 presents the traffic on the Ethernet interface. This interface is used only for accessing and checking the internal processes of the gateway, so the histograms depicted sporadically in the figure correspond to different such events. Figure 9 presents the traffic on the PPP interface. This is used for the communication of the gateway with the internet, so its traffic demonstrates a consistent pattern with some peaks when there is more activity in the gateway. Figure 10 presents the traffic on the WiFi interface. This is used only for accessing the gateway‘s web interface, so there is scattered traffic, while the peak at the end is related to the abnormal scenarios of the last day.

**Figure 11:**
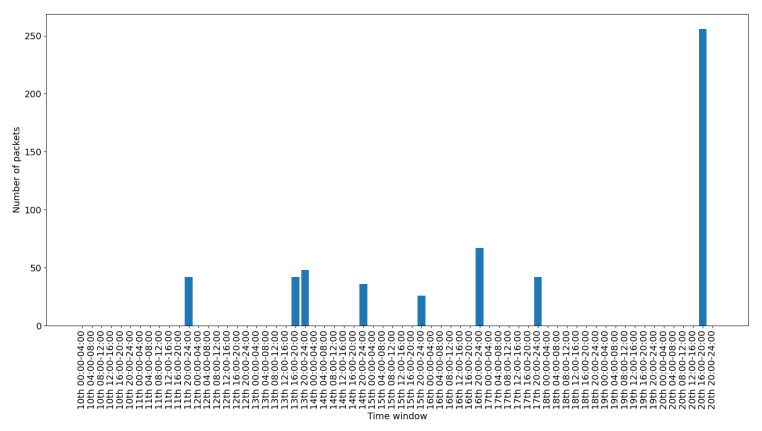
Bluetooth data packets time distribution.

**Figure 12:**
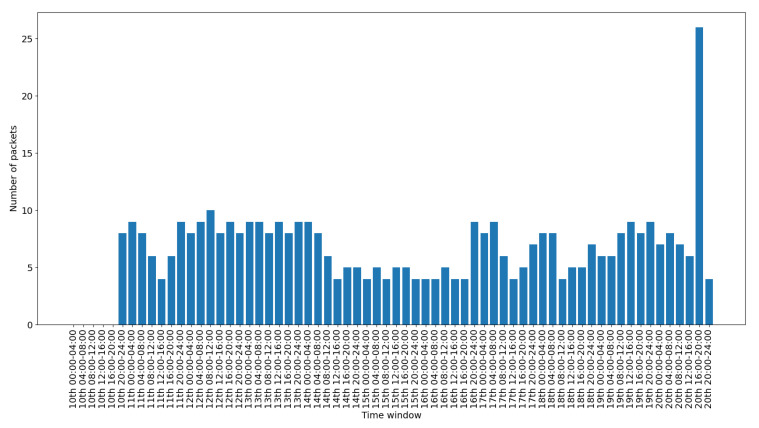
RF869 packets time distribution.

**Figure 13:**
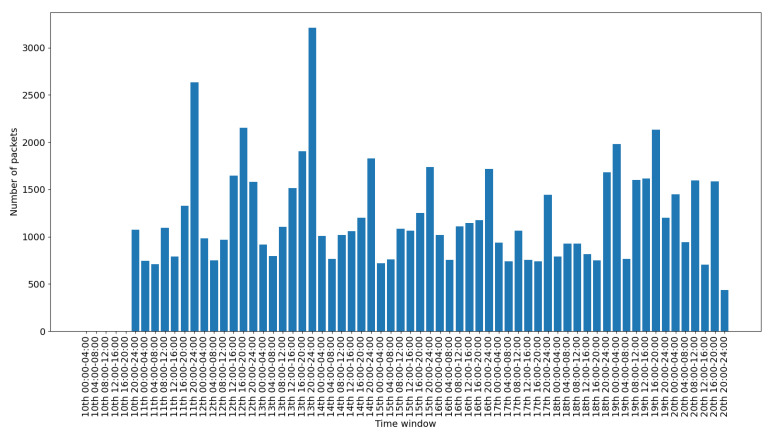
Zigbee packets time distribution.

Figure 11 presents the data traffic of the Bluetooth interface. Note that the management traffic of the gateway (which corresponds to much larger volume of traffic), has been filtered out for this illustration. The bars in the figure correspond to the occasional use of the Bluetooth devices, while the peak of the last day corresponds to the abnormal use scenario 2. Figure 12 presents the traffic on the RF869 interface. In this interface the single device connected, an emergency button, was only activated during the last day. The peak corresponds to the emergency button abnormal activation during scenario 3. Finally, Figure 13 presents the traffic on the Zigbee interface. The installed Zigbee devices are motion sensors and door contact sensors. The traffic rate seems to have a baseline corresponding to the packets exchanges with the gateway, irrespective of the activity in the smart-home. This is significantly increased for time slots at which the smart-home inhabitants are actively present in the home (typically between 20:00 and 24:00 everyday).

Overall, in an IoT system, the network traffic generated by the IoT devices follows a specific pattern. The traffic is triggered in the form of small standard chunks of packets by periodic functionality on environment events. Figure 8, Figure 9, Figure 10, Figure 11, Figure 12 and Figure 13 depict the number of packets during 4-h time-slots. It can be observed that the network traffic for each wireless interface exhibits a smooth behavior in the first nine days of the experiment when the smart-home system was operated under normal conditions. However, on the last day of the experiment, the statistics demonstrate an irregular peak due to the abnormal scenarios triggered on that day, meaning that the predictable patterns of the system’s normal status are disrupted when an anomaly happens within the system. A similar pattern can be observed to the other network traffic metrics as well, and this has been one of the main motivations behind designing and implementing NDFA to enhance smart-home security. Anomaly-based detection mechanisms can be implemented for the heterogeneous IoT environments based on the analysis of the normal and abnormal network patterns and thus to enable effective detection capabilities for secure systems.

Finally, it is noteworthy that the main advantage of the produced data-set is that it has been produced in real-world conditions. However, at the same time, this makes the calculation of data-set evaluation metrics not appropriate. A real-world data-set may not be characterized by statistically sound values for all the evaluation metrics, but this is expected due to the way it has been produced. Evaluation indices are suitable for evaluating artificial data-sets, in order to measure how realistic they are. On the contrary, real-world data-sets quality is based on the fact that they offer sufficiently high similarity between training data and live data for algorithms developed upon them.

## 7. Conclusions

The work presented in this paper is twofold as it has two main contributions: a traffic monitoring and analysis mechanism for smart-home environments and a real world IoT traffic data-set that can be used to develop anomaly detection mechanisms for IoT systems. Specifically, we propose an efficient scheme, that enables the extraction of significant data from traffic captures of multiple IoT protocols. The monitoring scheme developed in the GHOST project is hardware agnostic and can be applied in any smart-home installation. It is able to process network traffic on-line, extract meaningful information out of this traffic and facilitate the mitigation of detected abnormal situations. As far as we are aware, this is the first proposed mechanism that is capable of monitoring multiple IoT related protocols, given the restricted hardware resources of smart-home gateways. Thus, the performance comparison of our method to existing ones is not feasible, as no similar solutions exist.

Moreover, we produced and made available a ready to use IoT network traffic data-set. As a side contribution of our research and proof of concept of the implemented unified network traffic analysis mechanism, our paper provides the GHOST-IoT-data-set. This data-set is collected from a real-world smart-home set-up, with a rather diverse set of IoT devices, monitored for an extended period of time. To the best of our knowledge, this is the first smart-home traffic data-set containing data from multiple various wireless protocols on the same real-world installation. The compiled GHOST-IoT-data-set enabled the testing and evaluation of the GHOST modules which deal with anomaly detection and identification of abnormal incidents within a smart-home ecosystem [26]. Furthermore, it can be used for the validation of techniques focusing on the analysis of users’ normal and abnormal behavior, for anomaly detection of network traffic, detection of specific to IoT attacks, such as physical access attacks, and similar research efforts.

As future work, we also plan to create an additional data-set that will maintain the same quality features, such as real-world installation, multiple wireless interfaces and extensive capturing period, but will additionally contain more cyber-security related events. Thus, we intend to introduce more scenarios where the abnormal behavior is not due to accidental or intentional erroneous actions of the inhabitants but rather triggered by specific IoT based attacks launched against the devices or the gateway of the smart-home environment.

## Figures and Tables

**Table 1:** Devices deployed in the smart-home.

Device Type	Protocol	Placement	MAC
Motion sensor	ZigBee	Living room	1ba29f32
Motion sensor	ZigBee	Kitchen	f5df90f1
Motion sensor	ZigBee	Bathroom	73fce3d2
Motion sensor	ZigBee	Bedroom	a4b0d2cc
Door sensor	ZigBee	Entrance door	d5a41c16
Door sensor	ZigBee	Dishwasher	3c132b44
Emergency button	RF869	Nearby the gateway	00:05:BE
Weight scale	Bluetooth	Nearby the gateway	dab85476fc89
Blood pressure meter	Bluetooth	Nearby the gateway	8d944275c791
Gateway	Bluetooth	Office	cd19c93ef893
Gateway	ZigBee	Office	c08a1bf5
Smartphone	802.11 (WiFi)	Roaming around the house	e323b826aa71
Laptop	Ethernet	Nearby the gateway	ac756c521ced
Laptop	802.11 (WiFi)	Nearby the gateway	d96b0fddf228

**Table 2:** Events during capture period.

Day	Time	Event	Interface	Comments
10 October 2019	06:22:00 p.m.	Start capturing	–	–
11 October 2019	07:33:00 a.m.	Access to Care-life application using laptop	Ethernet	–
11 October 2019	07:38:00 a.m.	Placement of 802.15.4 sensor in final locations	–	–
–	–	Random accesses to Care-life app via smartphone to check the status	802.11	–
–	–	Normal activity of the devices	all	–
13 October 2019	08:00:00 p.m.	Connection through Eth interface	Ethernet	–
13 October 2019	08:03:00 p.m.	Copy of PCAP from SD to laptop by SSH	Ethernet	–
14 October 2019	08:23:00 p.m.	Restart of the Bedroom sensor	802.15.4	Removing and connecting the battery
15 October 2019	–	Not BLT measurements made this day	Bluetooth	User neglect it
16 October 2019	08:18:00 p.m.	Access through Ethernet to check and restart BLT process	Ethernet	–
17 October 2019	10:47:00 p.m.	Copy of PCAP from SD to laptop by SSH	Ethernet	–
20 October 2019	06:32:00 p.m.	Access through Ethernet to check the status	Ethernet	–
20 October 2019	06:35:00 p.m.	Scenario 1: Removal of the Living Room sensor’s battery	802.15.4	–
20 October 2019	06:36:00 p.m.	Scenario 2: 10 consecutive wrong blood pressure measures (without the required cuff)	Bluetooth	Erroneous measurements generate BLT traffic
20 October 2019	06:42:00 p.m.	Scenario 3: Emergency button activated 20 times in a row	RF869	–
20 October 2019	06:45:00 p.m.	Scenario 4: Entrance’s Door opening sensor uninstalled and many detections forced	802.15.4	–
20 October 2019	06:49:00 p.m.	Scenario 5: Removal of entrance door opening sensor’s the battery	802.15.4	–
20 October 2019	06:50:00 p.m.	Scenario 6: Relocation of the bedroom motion sensor to a nearer position	802.15.4	–
20 October 2019	07:06:00 p.m.	Scenario 7: Connection to the WiFi network from an unknown device (emulation of stolen passkey of the WiFi)	802.11	
20 October 2019	08:37:00 p.m.	Copy of PCAP from SD to laptop by SSH	Ethernet	–
20 October 2019	08:58:00 p.m.	System shut down and uninstall	–	–

**Table 3:** Numbers of packets/flows/batches per interface.

Interface	Number of Packets	Number of Flows/Batches	Avg Packets per Batch/Flow
Ethernet	36,360	311	116.9
Wireless	197,864	3399	58.2
PPP	2,953,647	485,649	6.1
Bluetooth	541,544	22,202	24.4
Zigbee	73,876	27,385	2.7
RF869	8128	8008	1.01

## Abbreviations

The following abbreviations are used in this manuscript:

AP	Access Point
BLE	Bluetooth Low Energy
C&C	Command and Control
DDoS	Distributed Denial of Service
GHOST	Safe-Guarding Home IoT Environments with Personalised Real-time Risk Control
IDS	Intrusion Detection System
IoT	Internet of Things
IP	Internet Protocol
LEACH	Low Energy Aware Cluster Hierarchy
NDFA	Network and Data Flow Analysis
PAN	Personal Area Network
PPP	Point-to-Point protocol
RPi	Raspberry Pi
RPL	Routing Protocol for Low-Power and Lossy Networks
TCP	Transmission Control Protocol
UDP	User Datagram Protocol
WEP	Wired Equivalent Privacy
WSN	Wireless Sensor Network
6LoWPAN	IPv6 over Low -Power Wireless Personal Area Networks
